# Supplementary material for: Mapping Immune Correlates and Surfaceome Genes in BRAF Mutated Colorectal Cancers
Source: Curr Oncol. 2023 Feb 21;30(3):2569–81. doi: 10.3390/curroncol30030196 (PMC10047091; doi:10.3390/curroncol30030196)
Supplement: Supplementary file 1 [file curroncol-30-00196-s001.zip › Supplementary Figures Caption.pdf]

Supplementary Figure S1. Bar graph that represents the expression of most upregulated genes (*AQP5, CD109, CLDN18, GP2, NTSR1, TMS4F4,* and *VNN1*) comparing samples from *BRAF* wt and mut COAD patients.

Supplementary Figure S2. Bar graph indicating the expression of most upregulated genes (*AQP5, CD109, CLDN18, GP2, NTSR1, TMS4F4,* and *VNN1*) comparing colorrectal *BRAF* wt and *BRAF* mut cell lines.

Supplementary Figure S3. Heatmap indicating the Rho and Spearsman's p-value of the correlation between the expression of upregulated genes (*CD74, FCGR2B, HLA-DMA, HLA-DMB, HLA-DOA, HLA-DPA1, HLA-DPB1, HLA-DQA1, HLA-DQB1, HLA-DRA, HLA-DRB1, HLA-DRB5,* and *LAG3)* and the infiltration level of some immune populations (CD8+ T cells, CD4+ T cells and myeloid dendritic cells (DC) in COAD (n=458) and READ (n=166).

Supplementary Figure S4. Dot plot showing the expression in READ (n=166) of upregulated genes (*CD74, FCGR2B, HLA-DMA, HLA-DMB, HLA-DOA, HLA-DPA1, HLA-DPB1, HLA-DQA1, HLA-DQB1, HLA-DRA, HLA-DRB1, HLA-DRB5,* and *LAG3*) in the Y axes and the infiltration level of some immune cells (CD8+ T, CD4+ T, and myeloids dendritic cells) in the X axes. The blue regression line shows the positive correlation and Rho and Spearsman's p-value data are presented in red.

Supplementary Figure S5. Heatmap indicating the Rho and Spearsman's p-value of the correlation between the expression of upregulated genes (*CD74, FCGR2B, HLA-DMA, HLA-DMB, HLA-DOA, HLA-DPA1, HLA-DPB1, HLA-DQA1, HLA-DQB1, HLA-DRA, HLA-DRB1, HLA-DRB5,* and *LAG3*) and the infiltration level of some immune population (CD8+ T cells, CD4+ T cells, myeloid dendritic cells, macrophages, and myeloid derived supressor cells (MDSC) in COAD (n=458).

Supplementary Figure S6. Bar graph that represents the expression of CD74 signature in relation to the status of MSI-H, comparing samples from CRC MSI low (0) and CRCMSI high (1). Mann-Whitney statistical p-value (p= 3.6E-06) was performed with a ± 95 % confidence interval.

Supplementary Figure S7. Bar graph that represents the expression of CD74 signature in each CRC subtypes (CMS1, CMS2, CMS3 and CMS4). Kruskal-Wallis statistical p-value was calculated (p=9.8E-28) with a ± 95 % confidence interval.

Supplementary Figure S8. Dot plot showing the expression in COAD (n=458) **(A)** and in READ (n=166) **(B)** of most upregulated genes (*AQP5, CD109, CLDN18, GP2, NTSR1, TMS4F4,* and *VNN1*) in the X axes and PD1 or PD(L)1 in the Y axes. The blue regression line shows the positive correlation and Rho and Spearsman's p-value data are presented in red.

Supplementary Figure S9. Heatmap representing the Rho and Spearsman's p-value of the correlation between the expression of most upregulated genes (*AQP5, CD109, CLDN18, GP2, NTSR1, TMS4F4,* and *VNN1*) and the infiltration level of some immune populations (CD8+ T cells, CD4+ T cells, myeloids dendritic cells, macrophages, and myeloid derived supresor cells) in COAD (n=458) and READ (n=166).

Supplementary Figure S10. Table that indicates the association between gene expression and tumor mutational burden where CD74 signature (in yellow) refers to the combined expression of: *CD74, FCGR2B, HLA-DMA, HLA-DMB, HLA-DOA, HLA-DPA1, HLA-DPB1, HLA-DQA1, HLA-DQB1, HLA-DRA, HLA-DRB1, HLA-DRB5,* and *LAG3,* while AQP5 signature (in green) refers to the combined expression of: *AQP5, CD109, CLDN18, GP2, NTSR1, TMS4F4,* and *VNN1* genes.
