# Supplementary material for: Mapping Immune Correlates and Surfaceome Genes in BRAF Mutated Colorectal Cancers
Source: Curr Oncol. 2023 Feb 21;30(3):2569–81. doi: 10.3390/curroncol30030196 (PMC10047091; doi:10.3390/curroncol30030196)
Supplement: Supplementary file 1 [file curroncol-30-00196-s001.zip › Supplementary Figures.pdf]

Supplementary Figure S1

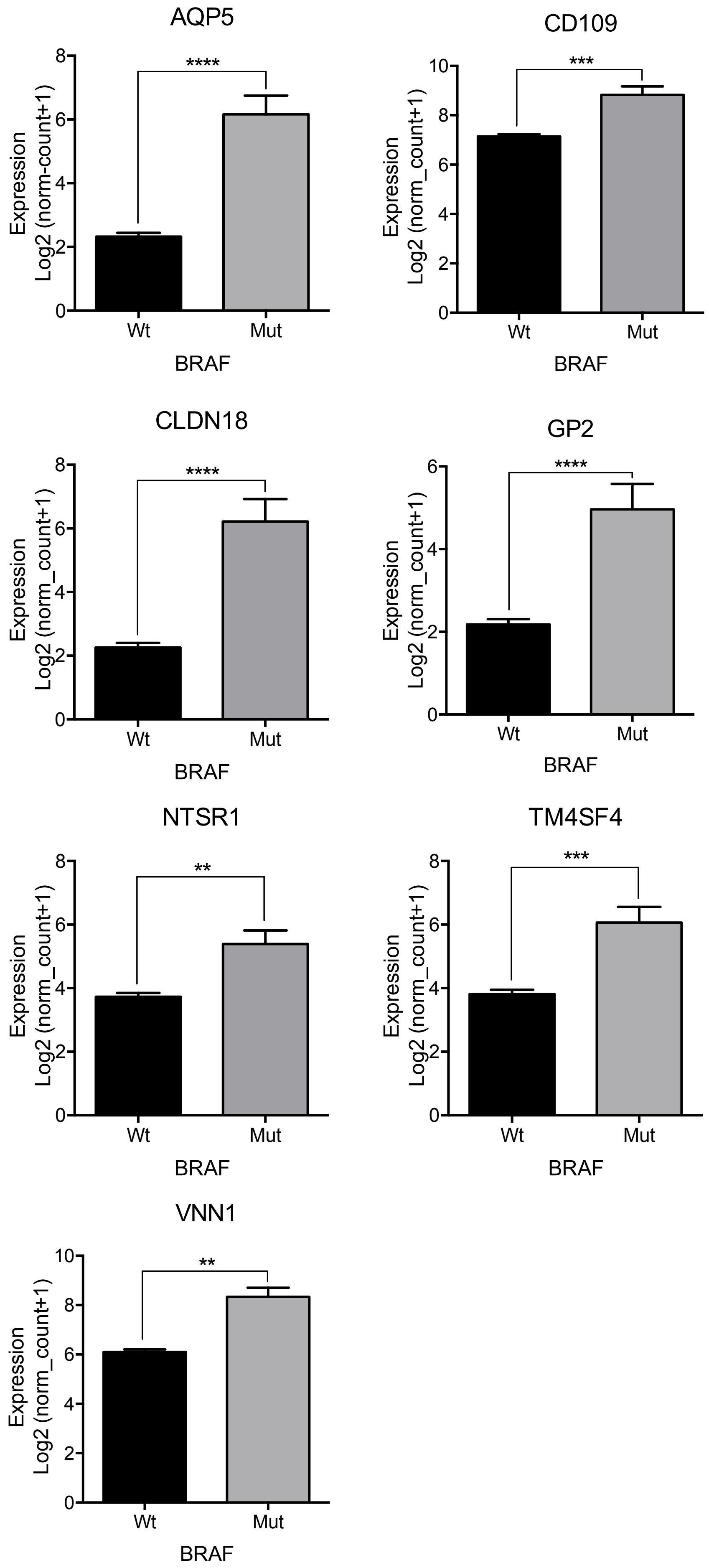

Supplementary Figure S2

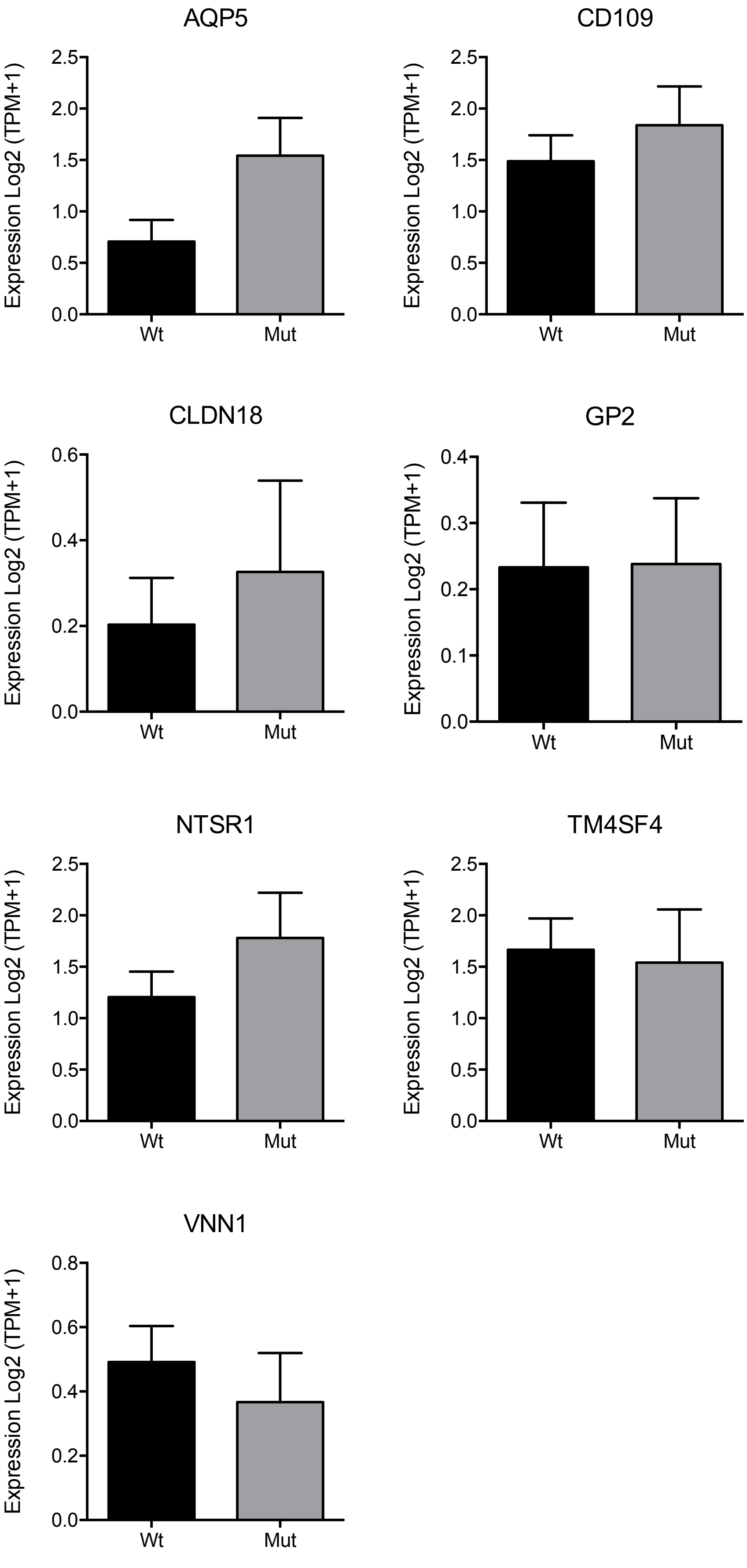

Supplementary Figure S3

	COAD (n=458)			READ (n=166)		
	T cell CD8+	T cell CD4+	DC	T cell CD8+	T cell CD4+	DC
CD74	Rho=0.521 p=1.64e-20	Rho=0.16 p=7.94e-03	Rho=0.717 p=1.13e-44	Rho=0.37 p=3.34e-04	Rho=0.198 p=6.09e-02	Rho=0.497 p=6.44e-07
FCGR2B	Rho=0.398 p=7.32e-12	Rho=0.246 p=3.74e-05	Rho=0.79 p=6.07e-60	Rho=0.36 p=4.84e-04	Rho=0.324 p=1.81e-03	Rho=0.732 p=2.36e-16
HLA-DMA	Rho=0.487 p=8.96e-18	Rho=0.127 p=3.48e-02	Rho=0.672 p=1.66e-37	Rho=0.281 p=7.29e-03	Rho=0.166 p=1.18e-01	Rho=0.416 p=4.54e-05
HLA-DMB	Rho=0.491 p=4.11e-18	Rho=0.152 p=1.14e-02	Rho=0.735 p=5.38e-48	Rho=0.364 p=4.14e-04	Rho=0.232 p=2.81e-02	Rho=0.581 p=1.86e-09
HLA-DOA	Rho=0.458 p=1.109e-15	Rho=0.31 p=1.53e-07	Rho=0.785 p=9.37e-59	Rho=0.393 p=1.29e-04	Rho=0.388 p=1.55e-04	Rho=0.746 p=3.19e-17
HLA-DPA1	Rho=0.516 p=4.17e-20	Rho=0.199 p=8.95e-04	Rho=0.798 p=6.84e-62	Rho=0.392 p=1.35e-04	Rho=0.32 p=2.09e-03	Rho=0.698 p=1.99e-14
HLA-DPB1	Rho=0.522 p=1.26e-20	Rho=0.234 p=9.12e-05	Rho=0.811 p=1.95e-65	Rho=0.353 p=6.41e-04	Rho=0.379 p=2.33e-04	Rho=0.727 p=4.78e-16
HLA-DQA1	Rho=0.435 p=4.14e-14	Rho=0.187 p=1.87e-03	Rho=0.739 p=8.52e-49	Rho=0.306 p=3.39e-03	Rho=0.18 p=9.03e-02	Rho=0.742 p=6.23e-17
HLA-DQB1	Rho=0.351 p=2.15e-09	Rho=0.014 p=8.2e-01	Rho=0.551 p=3.09e-23	Rho=0.201 p=5.75e-03	Rho=0.129 p=2.24e-01	Rho=0. 638 p=1.34e-11
HLA-DRA	Rho=0.532 p=1.83e-21	Rho=0.139 p=2.10e-02	Rho=0.767 p=1.54e-54	Rho=0.401 p=9.16e-05	Rho=0.212 p=4.5e-02	Rho=0.638 p=1.29e-11
HLA-DRB1	Rho=0.474 p=7.62e-17	Rho=0.067 p=2.71e-01	Rho=0.69 p=3.77e-40	Rho=0.299 p=4.16e-03	Rho=0.172 p=1.04e-01	Rho=0.691 p=4.6e-14
HLA-DRB5	Rho=0.454 p=2.15e-15	Rho=0.046 p=4.47e-01	Rho=0.631 p=5.81e-32	Rho=0.286 p=6.35e-03	Rho=0.217 p=3.96e-02	Rho=0.682 p=1.31e-13
LAG3	Rho=0.536 p=8.00e-22	Rho=0.173 p=4.10e-03	Rho=0.653 p=7.13e-35	Rho=0.363 p=4.30e-04	Rho=0.262 p=1.25e-02	Rho=0.542 p=3.47e-08

p<0.005, Rho >0.5

p<0.005, Rho= 0-0.5

p>0.005

p<0.005, Rho= 0-(-0.5)

p<0.005, Rho <-0.5

Supplementary Figure S4

READ (n=166)

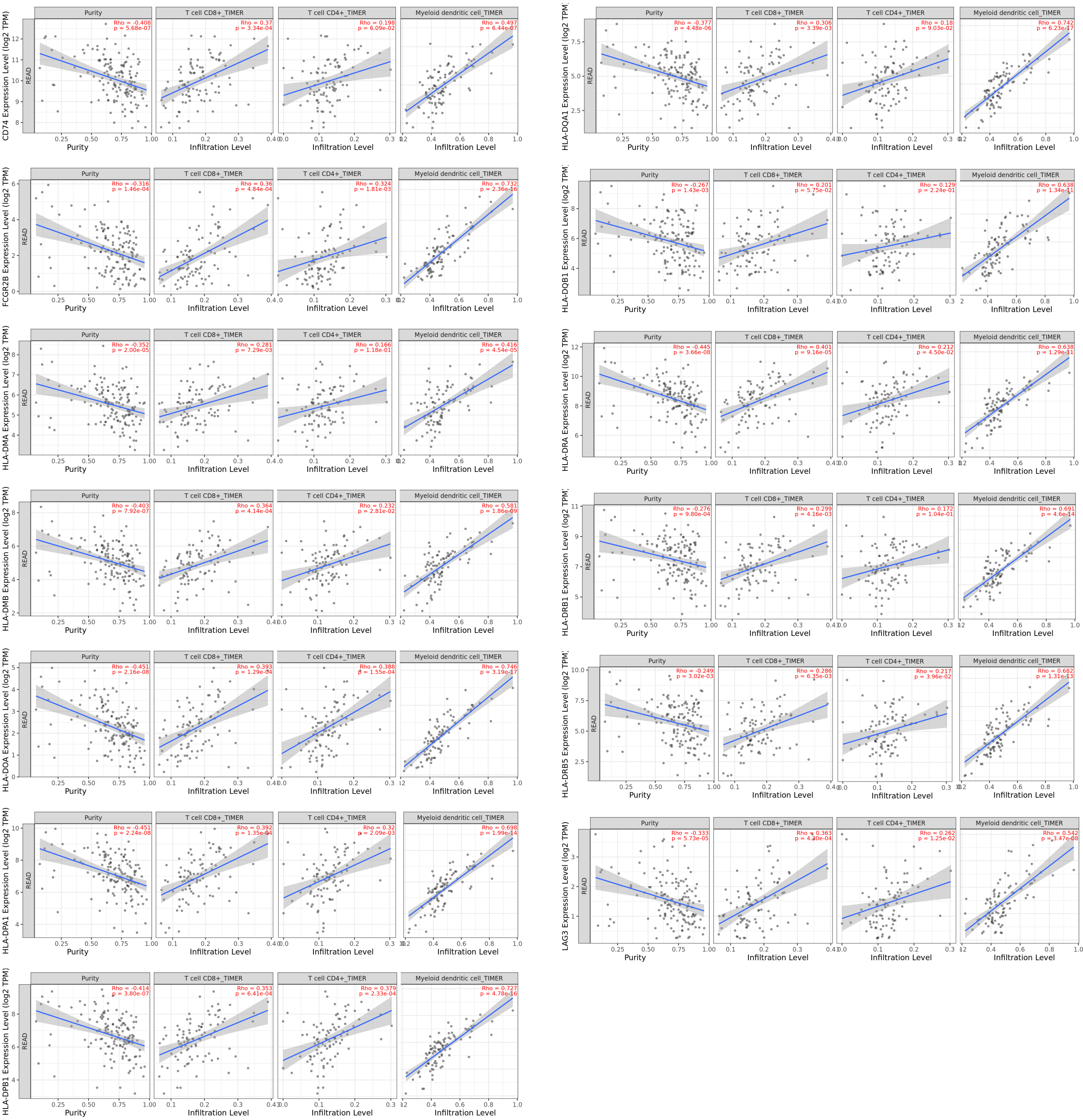

Supplementary Figure S5

COAD (n=458)	T cell $CD8^+$	T cell $CD4^+$	DC	Macroph.	MDSCs
CD74	Rho=0.521 p=1.64e-20	Rho=0.16 p=7.94e-03	Rho=0.717 p=1.13e-44	Rho=0.249 p=2.88e-05	Rho=-0.478 p=1.37e-24
FCGR2B	Rho=0.398 p=7.32e-12	Rho=0.246 p=3.74e-05	Rho=0.79 p=6.07e-60	Rho=0.546 p=8.65e-23	Rho=-0.634 p=5.15e-47
HLA-DMA	Rho=0.487 p=8.96e-18	Rho=0.127 p=3.48e-02	Rho=0.672 p=1.66e-37	Rho=0.231 p=1.13e-04	Rho=-0.427 p=2.12e-19
HLA-DMB	Rho=0.491 p=4.11e-18	Rho=0.152 p=1.14e-02	Rho=0.735 p=5.38e-48	Rho=0.311 p=1.47e-07	Rho=-0.542 p=2.00e-32
HLA-DOA	Rho=0.458 p=1.109e-15	Rho=0.31 p=1.53e-07	Rho=0.785 p=9.37e-59	Rho=0.418 p=4.41e-13	Rho=-0.593 p=6.29e-40
HLA-DPA1	Rho=0.516 p=4.17e-20	Rho=0.199 p=8.95e-04	Rho=0.798 p=6.84e-62	Rho=0.382 p=5.38e-11	Rho=-0.570 p=2.52e-36
HLA-DPB1	Rho=0.522 p=1.26e-20	Rho=0.234 p=9.12e-05	Rho=0.811 p=1.95e-65	Rho=0.428 p=1.18e-13	Rho=-0.647 p=1.55e-49
HLA-DQA1	Rho=0.435 p=4.14e-14	Rho=0.187 p=1.87e-03	Rho=0.739 p=8.52e-49	Rho=0.419 p=4.27e-13	Rho=-0.626 p=1.40e-45
HLA-DQB1	Rho=0.351 p=2.15e-09	Rho=0.014 p=8.2e-01	Rho=0.551 p=3.09e-23	Rho=0.21 p=4.44e-04	Rho=-0.434 p=4.45e-20
HLA-DRA	Rho=0.532 p=1.83e-21	Rho=0.139 p=2.10e-02	Rho=0.767 p=1.54e-54	Rho=0.303 p=2.98e-07	Rho=-0.536 p=1.32e-31
HLA-DRB1	Rho=0.474 p=7.62e-17	Rho=0.067 p=2.71e-01	Rho=0.69 p=3.77e-40	Rho=0.269 p=6.25e-06	Rho=-0.528 p=1.70e-30
HLA-DRB5	Rho=0.454 p=2.15e-15	Rho=0.046 p=4.47e-01	Rho=0.631 p=5.81e-32	Rho=0.174 p=3.70e-03	Rho=-0.478 p=1.58e-24
LAG3	Rho=0.536 p=8.00e-22	Rho=0.173 p=4.10e-03	Rho=0.653 p=7.13e-35	Rho=0.194 p=1.20e-03	Rho=-0.403 p=2.73e-17

- p<0.005, Rho >0.5
- p<0.005, Rho= 0-0.5
- p>0.005
- p<0.005, Rho= 0-(-0.5)
- p<0.005, Rho <-0.5

Supplementary Figure S6

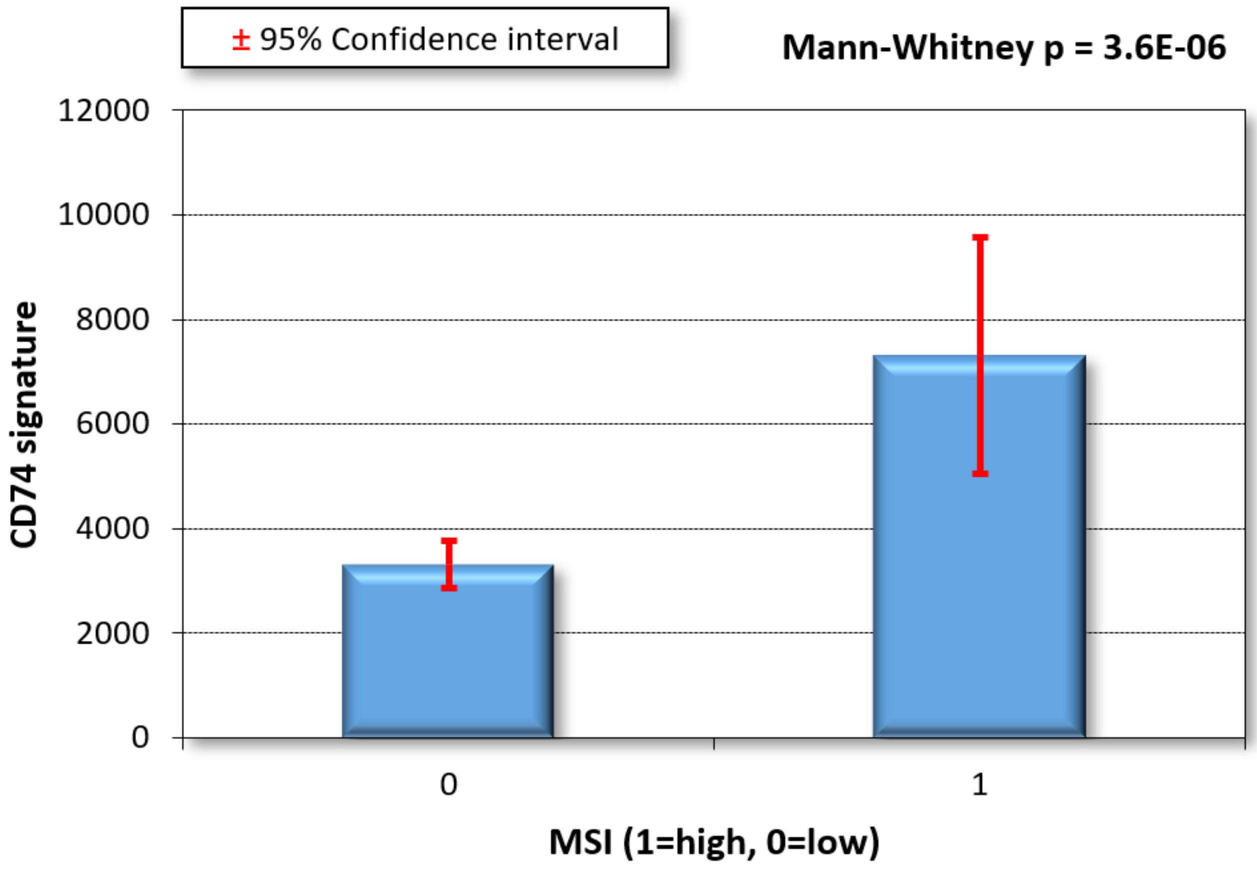

Supplementary Figure S7

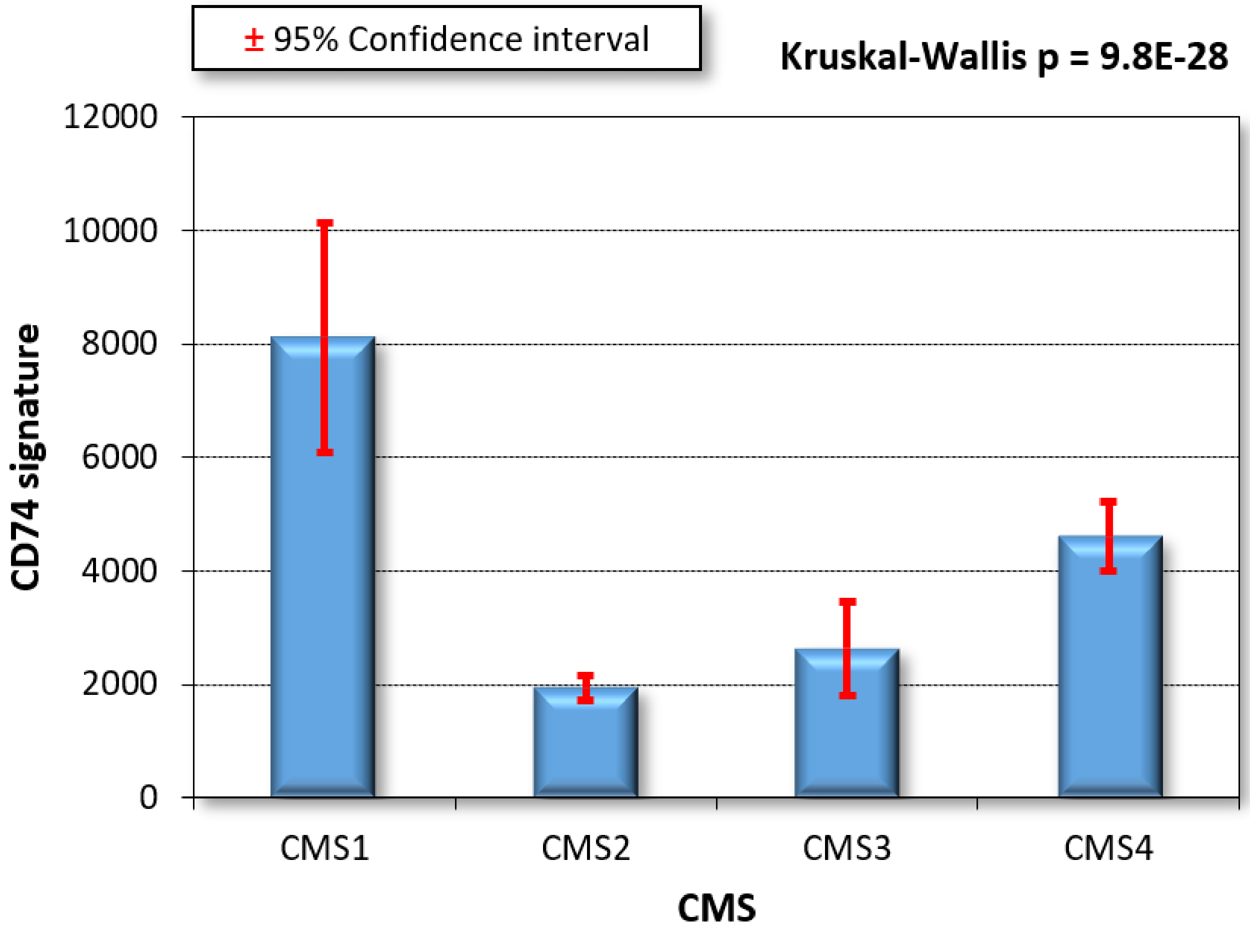

Supplementary Figure S8

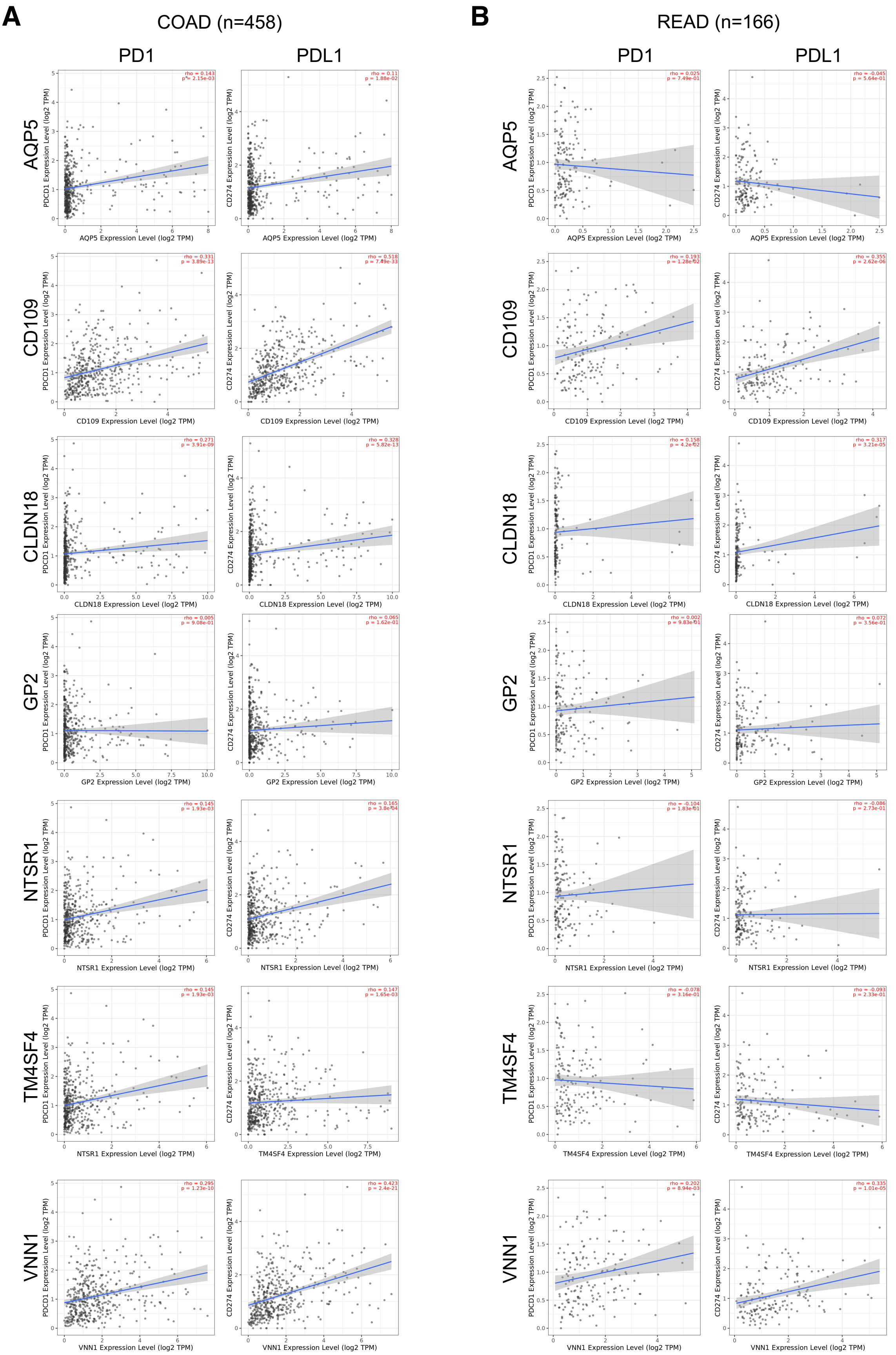

Supplementary Figure S9

	COAD (n=458)				
	T cell CD8+	T cell CD4+	DC	Macroph.	MDSCs
AQP5	Rho=0.152<br>p=1.14e-2	Rho=-0.058<br>p=3.34e-01	Rho=0.117<br>p=5.26e-02	Rho=-0.08<br>p=1.86e-01	Rho=0.09<br>p=6.86e-02
CD109	Rho=0.319<br>p=6.32e-08	Rho=0.185<br>p=2.08e-03	Rho=0.617<br>p=3.22e-30	Rho=0.45<br>p=3.78e-15	Rho=-0.101<br>p=4.10e-02
CLDN18	Rho=0.321<br>p=5.09e-08	Rho=-0.019<br>p=7.49e-01	Rho=0.319<br>p=6.33e-08	Rho=0.018<br>p=7.66e-01	Rho=-0.082<br>p=9.86e-02
GP2	Rho=0.045<br>p=4.55e-01	Rho=-0.065<br>p=2.86e-01	Rho=-0.069<br>p=2.52e-01	Rho=-0.06<br>p=3.22e-01	Rho=-0.026<br>p=6.07e-01
NTSR1	Rho=0.159<br>p=8.12e-03	Rho=0.054<br>p=3.69e-01	Rho=0.244<br>p=4.30e-05	Rho=0.216<br>p=3.06e-04	Rho=-0<br>p=9.97e-01
TM4SF4	Rho=0.206<br>p=6.01e-04	Rho=0.199<br>p=8.95e-04	Rho=0.16<br>p=8.00e-03	Rho=0.06<br>p=3.21e-01	Rho=-0.086<br>p=8.25e-02
VNN1	Rho=0.378<br>p=9.16e-11	Rho=-0.105<br>p=8.20e-02	Rho=0.32<br>p=5.97e-08	Rho=-0.006<br>p=9.21e-01	Rho=-0.247<br>p=4.90e-07

	READ (n=166)				
	T cell CD8+	T cell CD4+	DC	Macroph.	MDSCs
AQP5	Rho=-0.204<br>p=5.41e-02	Rho=0.12<br>p=2.60e-01	Rho=-0.274<br>p=9.07e-03	Rho=0.028<br>p=7.96e-01	Rho=0.139<br>p=1.03e-01
CD109	Rho=0..278<br>p=8.02e-03	Rho=0.404<br>p=7.84e-05	Rho=0.53<br>p=7.65e-08	Rho=0.446<br>p=1.06e-05	Rho=0.054<br>p=5.24e-01
CLDN18	Rho=0.021<br>p=8.46e-01	Rho=0.019<br>p=2.62e-01	Rho=0.035<br>p=7.41e-01	Rho=0.0.57<br>p=5.94e-01	Rho=0.03<br>p=7.29e-01
GP2	Rho=-0.056<br>p=5.99e-01	Rho=-0.085<br>p=4.24e-01	Rho=-0.067<br>p=5.31e-01	Rho=-0.059<br>p=5.81e-01	Rho=-0.105<br>p=2.17e-01
NTSR1	Rho=-0.009<br>p=9.31e-01	Rho=0.012<br>p=9.08e-01	Rho=-0.089<br>p=4.05e-01	Rho=0.008<br>p=9.38e-01	Rho=-0.079<br>p=3.54e-01
TM4SF4	Rho=-0.054<br>p=-6.12e-01	Rho=0.32<br>p=2.09e-03	Rho=0.16<br>p=8.84e-01	Rho=0.116<br>p=2.77e-01	Rho=-0.006<br>p=9.45e-01
VNN1	Rho=0.239<br>p=2.31e-02	Rho=0.067<br>p=5.31e-01	Rho=0.171<br>p=1.08e-01	Rho=0.168<br>p=1.14e-01	Rho=-0.347<br>p=2.85e-05

- p<0.005, Rho >0.5
- p<0.005, Rho= 0-0.5
- p>0.005
- p<0.005, Rho= 0-(-0.5)
- p<0.005, Rho <-0.5

Supplementary Figure S10

	CD74 signature	AQP5 signature
**Number of Immunogenic Mutation**		
Correlation coefficient	0.233	0.219
Valid cases	390	390
One-sided significance	1.6E-6	6.3E-06
**Number of Binding Expressed PMHC**		
Correlation coefficient	0.267	0.244
Valid cases	390	390
One-sided significance	4.1E-08	5.3E-07
**Total Number of Mutations**		
Correlation coefficient	0.193	0.230
Valid cases	412	412
One-sided significance	3.9E-05	1.1E-06
